# Optimization of a mAb production process with regard to robustness and product quality using quality by design principles

**DOI:** 10.1002/elsc.202100172

**Published:** 2022-06-03

**Authors:** Ole Jacob Wohlenberg, Carlotta Kortmann, Katharina V. Meyer, Jana Schellenberg, Katharina Dahlmann, Janina Bahnemann, Thomas Scheper, Dörte Solle

**Affiliations:** ^1^ Institut für Technische Chemie Leibniz Universität Hannover Hannover Germany

**Keywords:** ambr®, chinese hamster ovary, monoclonal antibody, process analytical technology, quality by design

## Abstract

Quality by Design principles are well described and widely used in biopharmaceutical industry. The characterization of a monoclonal antibody (mAb) production process is crucial for novel process development and control. Yet, the application throughout the entire upstream process was rarely demonstrated. Following previously published research, this study marks the second step toward a complete process characterization and is focused on the effect of critical process parameters on the antibody production efficiency and quality of the process. In order to conduct the complex Design of Experiments approach with optimal control and comparability, the ambr®15 micro bioreactor platform was used. Investigated parameters included the pH and dissolved oxygen set points, the initial viable cell density (iVCD) as well as the N‐1 duration. Various quality attributes (e.g., growth rate, viability, mAb titer, and peak proportion) were monitored and analyzed using multivariate data analysis to evaluate the parameter effects. The pH set point and the initial VCD were identified as key process parameters with strong influence on the cell growth as well as the mAb production and its proportion to the total protein concentration. For optimization and improvement in robustness of these quality attributes the pH must be increased to 7.2, while the iVCD must be lowered to 0.2 × 10^6^ cells/mL. Based on the defined design space, additional experiments verified the results and confirmed the intact bioactivity of the antibody. Thereby, process control strategies could be tuned toward high cell maintenance and mAb production, which enable optimal downstream processing.

**Abbreviations**
CQAs: critical quality attributes
DoE: design of experiments
EDV: ebola virus disease
mAb: monoclonal antibody
PAT: process analytical technology
QbD: quality by design

## 1 INTRODUCTION

The importance of monoclonal antibodies in the biopharmaceutical industry is continuously growing, with past market data indicating an increasing trend for 2022 and overall mAb sales reaching 130–200 billion US$. [1] Current use cases and studies also underline the treatment possibilities of mAbs for novel diseases such as the Ebola Virus Disease (EVD) or the Severe Acute Respiratory Syndrome CoronaVirus‐2 (SARS‐CoV‐2). [2, 3, 4] Rapid product and process development with a controlled production chain ensuring highest quality standards are necessary to enable fast approval and distribution of new therapeutic antibodies.

Therefore, the FDA launched a guidance protocol introducing the Quality by Design (QbD) approach, a risk‐based workflow to biopharmaceutical product development and manufacturing. [5] Said protocol was based on the *Current Good Manufacturing Practice for the 21^st^ century* initiative and consist of a framework for Process Analytical Technology (PAT) and multiple guidelines from the International Conference of Harmonisation (ICH). [6, 7, 8, 9, 10]

Until then, the development and manufacturing were limited by inflexible batch to batch quality controls as well as an unstructured relation between the process and the product application. The QbD approach aims to systematically improve a process toward product quality, regulatory compliance, cost reduction and fast track development. [11] In a split up approach individual process steps are investigated separately with specific intermediate quality outputs, before the gained results and process knowledge can be combined in a holistic way for the entire mAb production process. Therefore, the key objective of QbD with said method is to identify the intermediate critical quality attributes for the process, which can influence the products critical quality attributes (CQAs) as well as critical process parameters (CPPs) in order to establish a designated design space for the studied process. [12, 13]

The targeted roadmap for QbD implementation in the process development begins with a risk assessment, mostly using screening experiments and the Failure Mode and Effect Analysis (FMEA) approach. [14] Parameters considered as critical for the process stability or product quality are further investigated in a Design of Experiments (DoE). This enables a structured connection between process in‐ and outputs to identify optimal process conditions for the predetermined targets, conclusively resulting in the design space. [15, 16]

**FIGURE 1:**
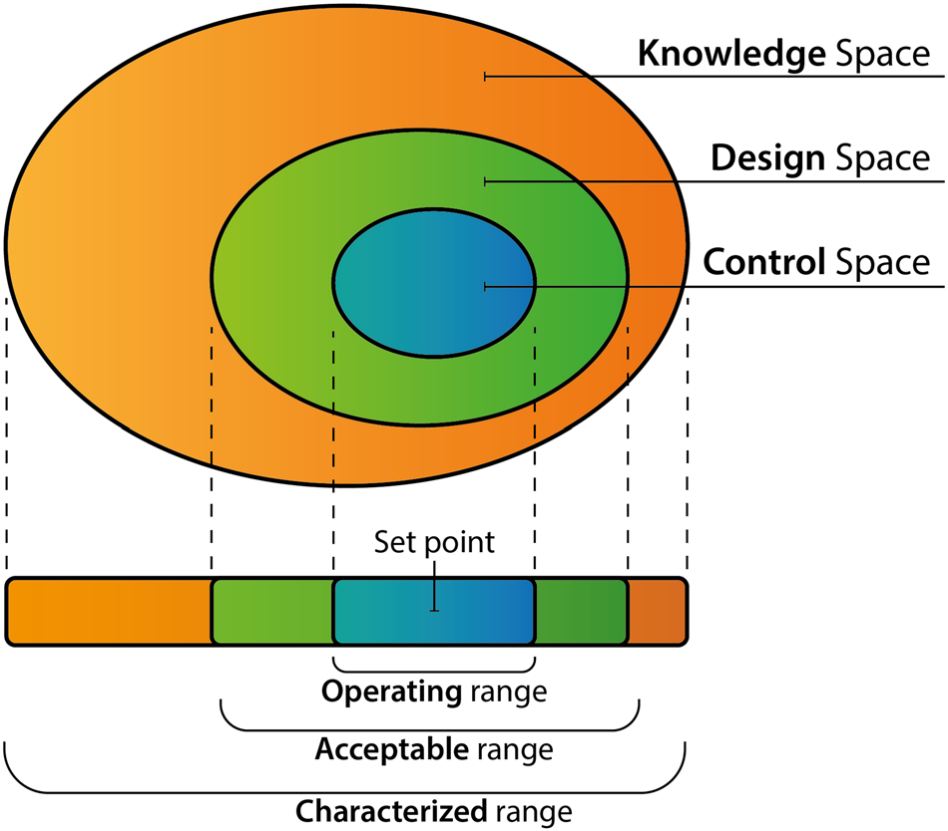
Schematic representation of the design space and associated ranges. Adjusted from Rathore et al. [17]

A design space represents the multidimensional connection and interaction of process factors that assure a robust process operation and observance of CQAs. [7] Thereby, working within the factorial boundaries of the design space is not considered to be a change or risk for the conducted process, enabling a more flexible, cost saving, and steady workflow. The relationship of the design space with the characterized knowledge space and the control space with their associated factor ranges is depicted in Figure 1.

In this work, the main focus will be set on important intermediate process quality attributes like cell growth and viability as well as the mAb production efficiency and quality. In order to ensure sufficient product quality, the mAb proportion to total protein concentration and the bioactivity will be examined as indicators for the target product profile. Thereby, an optimization of the established process regarding the product yield with sufficient bioactivity can be achieved within the described QbD guidelines. Combination of these intermediate quality attributes will lead to the establishment of a designated design space for the production process.

Based on previous inoculum expansion studies, this marks the second step toward a complete process characterization. [18] While some case studies for QbD in mAb productions were conducted, the full process implementation is important for every novel biopharmaceutical product as well as a basis to gain general process knowledge and confidence in the presented QbD tools. [19] One major challenge toward this goal is the long process duration for Chinese Hamster Ovary (CHO) cell cultivations and the amount of experiments needed for a sufficient DoE and the subsequent modeling. In order to overcome this, while maintaining optimal process control and comparability, the experiments were conducted using the ambr®15 micro bioreactor system. [20, 21] These small‐scale bioreactors are commonly used a scale‐down model for fed batch processes and enable the parallel control of up to 24 cultivations. Thereby, a more rapid application of QbD strategies for the production process is possible.

## 2 MATERIAL AND METHODS

The presented QbD principles will be implemented in the production step of an IgG1 monoclonal antibody (mAb) production process using a DG44 CHO cell line (Sartorius Stedim Cellca GmbH).

### 2.1 Cell line and material

The inoculum expansion was performed as described by Boehl et al. [18] For the production process the cells were cultivated in the Ambr®15 system (Sartorius, Germany). The cultivation was conducted in proprietary and chemically defined cell culture media, all part of the Sartorius Stedim Cellca medium platform. Production medium (PM) and two additional feed media (feed medium A; feed medium B) for macro nutrients (e.g., glucose) and micro nutrients (e.g., amino acids) respectively, were used for the production process. [18, 21]

The cultivation was conducted over 11 days with daily feeds (1 % feed medium A, 0.1 % feed medium B) from day 3 and additional glucose feeds to a culture glucose concentration of 5 g/L from day 5. Feeding volume for needed concentrations was calculated by the ambr®15 software.

### 2.2 Analytics

Samples were taken daily by the Ambr®15 liquid handler during the production process. Viable cell densities and viabilities were measured using a Cedex HiRes (Roche Innovatis, Switzerland) with a 1:2 dilution in 10% phosphate‐buffered saline (PBS). Growth rates were calculated by linear regression of the natural logarithm of viable cell densities against culture duration. The pH and pO_2_ were measured by the Ambr®15 system. Offset calibrations for the PH were performed using a FiveEasy Plus pH meter FP20‐Micro (Mettler Toledo, USA) every two days.

The mAb titer and important substrates (e.g., glucose; amino acids) were analyzed during the production process using the Cedex Bio (Roche, Swiitzerland). Therefore, cell separation was performed by centrifugation for 5 min at 190 × *g* using a Microstar17 centrifuge (VWR International, USA). Specific productivities were calculated by dividing the final antibody titer by the integral viable cell concentration over the entire production process duration. The total protein concentration of the supernatant was analyzed by high‐performance liquid chromatography (HPLC) as described by Meyer et al. and used for calculation of the mAb peak proportions. [22]

**PRACTICAL APPLICATION**

The ambr®15 micro bioreactor platform was used to study the production process of a monoclonal antibody within a complex Design of Experiments approach. Our controlled investigation of critical process parameters resulted in the establishment of a designated design space and process control strategies for robust process optimization. The gained process understanding and novel feedback strategies are crucial for the optimization of the studied process as well as for new process development. Thereby, this study highlights the importance of Quality by Design and the practical implementation using micro bioreactors.

Antibody bioactivity was determined by an adherent mouse fibroblast (L929) cell based assay using the tumor necrosis factor alpha (TNF‐α) under the presence of actinomycin D. [23] Therefore, L929 cell viability was analyzed using the cell titer‐blue assay (Promega, USA) after 24 h of antigen/antibody treatment. The produced antibody was diluted and used in low and high concentrations of 2.5 and 50 ng/mL respectively. The antigen TNF‐α was used in a fixed concentration, determined to result in around 30% cell viability.

### 2.3 Failure mode and effect analysis (FMEA)

The risk assessment was conducted using the FMEA approach as described by Boehl et al., with five levels for the classification of the probability, severity and detectability of each process parameter. [18] Multiplication of these rated factors resulted in the respective risk priority number. The Probability (P) evaluates the likelihood of general parameter failure. Failure impact on the system and the product are assessed by the Severity (S). In addition, the Detection (D) assesses the possibility of detection and fast correction a given failure mode.

### 2.4 Design of Experiments (DoE)

The design and analysis was performed using the DoE software MODDE 12 (Umetrics, Sartorius Stedim Data Analytics, Germany). The four parameters with the highest RPN during the risk assessment were used as factors (F_1 _= pH, F_2 _= initial viable cell density, F_3 _= N‐1 duration, F_4 _= pO_2_) for a custom central composite face centered design with three center point runs. The resulting design is illustrated in Figure 3 (see chapter ‘DoE structure and implementation’). Hereinafter, the different factor settings are described as 0 for center point level and –1/1 for the low and high levels of the full factorial cube respectively. The additional levels for the 5 level factor (initial VCD) are described as –2 and 2.

The pH was varied equally between 6.9 and 7.3 by control of the CO_2_ gas flow and additional bolus feeds of sodium carbonate (Na_2_CO_3_) as base. For the initial viable cell density 5 levels from 0.1 to 0.5 × 10 [6] cells/mL with an equidistant step size of 0.1 × 10 [6 cells/mL were examined. The N‐1 duration was varied equally between 2 and 4 days by shifting of the passage times of the N‐2 and N‐3 passage to allow simultaneous inoculation of the production process. The pO_2_ was controlled over the oxygen gas flow and varied equally between 40% and 80%. The applied design resulted in a total of 33 runs. The experiments were conducted in two Ambr15 cultivations with 18 vessels each. Three center point runs were implemented in both cultivations, resulting in a total of 36 conducted runs. An overview of the experimental set up and the explained numerical coding of the parameter levels are depicted in the supplements.

Six different responses were analyzed: growth rate for the growth phase during the production process, integral viable cell concentration, end viability, total mAb titer, mAb productivity, and the mAb peak proportion. The responses were predicted by using a multilinear model with squares and interactions for all factors.

Multiple linear regression (MLR) was used to fit the mathematical models as described by Boehl et al. [18] Model statistics such as the R‐squared, adjusted R‐squared, Q‐squared, model validity, and the reproducibility were analyzed for the evaluation of the conducted model. Factors whose coefficient has the value zero in its confidential interval are regarded to have no significant influence on the response and are therefore removed from the model.

The design space was calculated using Monte Carlo simulations to compile the needed probability statistics. Parameter limits are summarized in the supplements and were set on the basis of historical data and process knowledge.

## 3 RESULTS AND DISCUSSION

Main objective of this study was the investigation of parameter effects and the establishment of a designated design space for the mAb production process of a CHO cell cultivation. Therefore, process parameters were identified and assessed by their theoretical risks. Identified critical process parameters were evaluated based on a design of experiment approach with a focus on cell growth, mAb production and the mAb proportion to process related impurities as intermediate critical quality attributes.

### 3.1 Risk assessment

**FIGURE 2:**
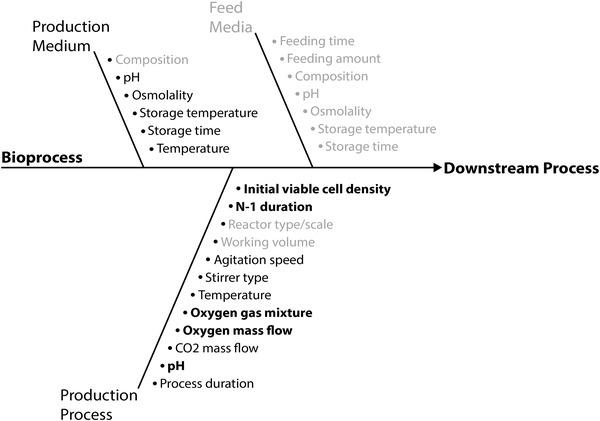
Ishikawa diagram for the mAb production process. Bold parameters were determined as critical and therefore examined during this study. Grey parameters were excluded from the risk assessment

An Ishikawa diagram (Figure 2) was used to list possible process parameters, categorized in different parts of the process, namely the medium, feeding, process and the cell separation. These parameters can influence the process and can therefore be potential root causes for specific failure events. In order to rank the process parameters according to said risks, a Failure Mode and Effect Analysis approach was conducted.

Parameters that were highly optimized during the process development, like the media composition of the production medium and the feeding, were not taken into account because these parameters are fixed for the production process and not part of the process control. Previous scale down experiments showed high process reproducibility in various reactor systems and scales, which is why the reactor scale and working volume were also considered noncritical parameters. Parameters, like the temperature or feeding amounts/timing, are highly controlled in the ambr®15 system. This results in a low failure probability and a quick detection of potential failure modes like temperature drops. In order to determine CPPs the RPN threshold was set to be over 20, which represents moderate to significant risk (3–5) of at least two factors with the third factor having any considerable risk (>1) during the FMEA. The results of the FMEA for the identified CPPs are listed in Table 1 and shown bold in Figure 2.

**TABLE 1:** Failure mode and effect analysis (FMAE) results for the examined parameters. Probability, severity and detection were individually rated between 1 and 5. 1 represents no potential risk, 3 moderate/controllable risk and 5 significant risk

Process parameter	Probability	Severity	Detection	RPN
pH	2	5	3	30
Initial VCD	2	3	4	24
Dissolved oxygen	3	4	2	24
N‐1 duration	2	3	4	24
Process duration	2	5	2	20
Media storage temperature	1	4	3	12
CO_2_ mass flow	2	3	2	12
Temperature	1	5	2	10
Media storage time	1	4	2	8
Agitation speed	1	4	2	8
Stirrer type	1	3	1	3

The pH value of the production medium was used as factor F_1_ in the DoE due to its severe effect on the cell growth as well as the antibody production and stability (Severity = 5). As a result of the high level of monitoring and control, the probability of larger pH failures can be considered fairly small (Probability = 2). Yet, control systems like base additions work with a time delay and can add to additional cell stress (Detection = 3).

Significance of the initial VCD (F_2_) can be explained by moderate risk of all three FMEA factors. A moderate impact on the growth rate was shown in previous preculture experiments (Severity = 3). This effect was correlated to higher nutrient consumption rates and thus a nutrient deficiency during first days of the cultivation. The possibility for failures in the iVCD was considered to be low yet possible, due to potential inaccuracies during the inoculation and rare variations in the preculture cell density (Probability  =  2). Deviations of the initial VCD can be detected quickly; however, readjustments can potentially lead to a critical process delay. In biopharmaceutical production scale the readjustment to the VCD set‐point can furthermore be virtually impossible, due to the preparation and availability of bioreactors (Detection  =  4).

In contrast, failures in the dissolved oxygen (F_3_) can be detected and corrected quite quickly (Detection = 2). However, oxygen plays an important role in the cell metabolism and was therefore considered critical for cell growth and production (Severity = 4), while historical data showed a moderate probability of slight failure modes (Probability = 3).

The passage duration was previously determined as a critical process parameter during the inoculum expansion study and showed negative effects on the cell growth and viability with longer durations. [18] During said study, the N‐1 passage was set to be 3 days for all experiments to enable simultaneous inoculation of the production process with constant volumes. In order to study the link between critical process parameters of the inoculum expansion and the production process, the N‐1 duration was used as another factor F_4_ for the DoE. Even though the probability of longer N‐1 durations can be consider fairly small (Probability  =  2), a process delay can have a critical impact on the cell conditions, growth and viability (Severity = 3). Moreover, the detection must be assessed as critical (Detection = 4), due to the time delay for the process and inability to solve certain failure modes, without preparing another bioreactor.

### 3.2 DoE structure and implementation

**FIGURE 3:**
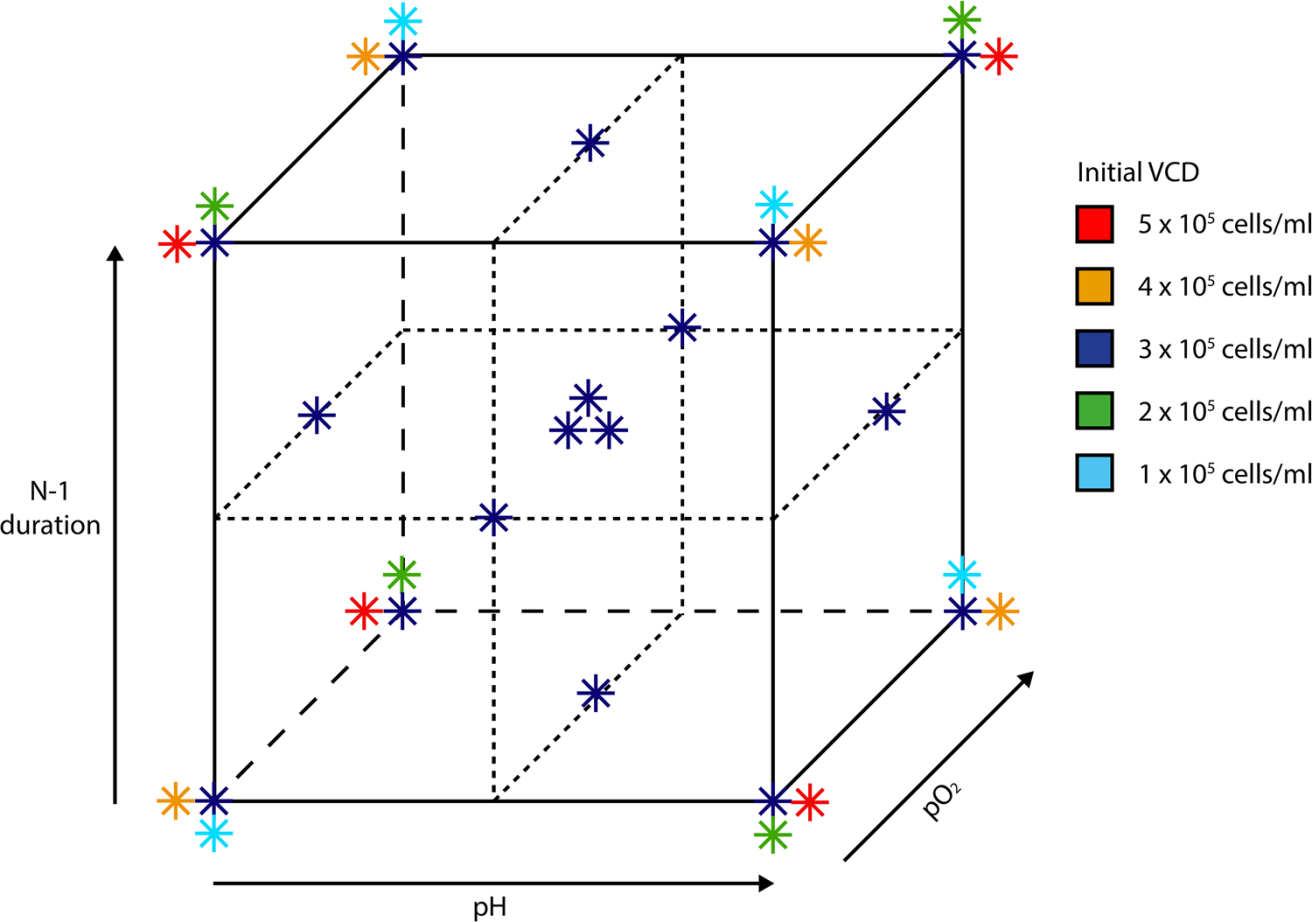
Schematic representation of the conducted design of experiments for the N‐1 duration, pH, dissolved oxygen (pO_2_) and the initial VCD. The corner point stars of the cube represent –1/1 levels of the studied parameters. The three stars in the middle of the cube represent the replicated center point runs (0 level). The color of the stars represent the 5 levels of the initial VCD (–2/–1/0/1/2 level)

In order to investigate the influence and interaction of the determined critical process parameters, based on the ICH Guidelines, a Design of Experiments was set up. Since four parameters, namely the pH (F_1_); initial VCD (F_2_); Dissolved oxygen (F_3_) and N‐1 duration (F_4_), exceeded the critical RPN threshold, a custom extended central composite design was constructed. Therefore, the factors F_1_, F_3_ and F_4_ were combined in a face centered design and each varied on a three level scale (–1, 0, 1). The initial VCD (F_2_) was varied on a five level scale (–2, –1, 0, 1, 2) in order to expand the experimental range and integrated in the design as a fractional factorial factor. The resulting design, depicted in Figure 3, shows a regular geometry and high coverage for all four critical process parameters.

The design added up to 33 experimental runs. In order to extract in‐depth information about the varied process parameters a selection of critical responses for the studied process was determined. During the production process the growth rates and integral viable cell concentration of each run were monitored as responses for cell growth and maintenance. The proliferation rate is a critical attribute correlated to the cell condition and can provide early information about possible delays in the culture duration. Furthermore, general improvements in cell growth could lead to reduced process time and production costs. The same argumentation applies for a high integral viable cell concentration, since it indicates optimal cell growth and maintenance conditions.

Additionally, the end viabilities were determined to further investigate the cellular conditions. High cell viabilities throughout the process also insures minimal amounts of cell debris, nucleic acids and host cell proteins, which have to be removed expensively during the downstream process. In order to investigate quality attributes for the mAb production, the total mAb titers were determined at the end of the cultivations, which has to be maximized for a profitable process. The specific productivity was determined to elucidate the cell specific productivity for all studied conditions, since earlier experiments showed a contrary trend between optimal growth and high specific productivity. Furthermore, the mAb peak proportion was analyzed as a quality attribute for all runs. The proportion of produced antibody to host cell proteins and other impurities directly impacts the following downstream and possible yields.

The summary of all experimental runs with associated responses is depicted in the supplements. By combination of said responses, the optimal process conditions for maximized growth and cell maintenance as well as optimal mAb production could be determined.

### 3.3 Multivariate data analysis

**FIGURE 4:**
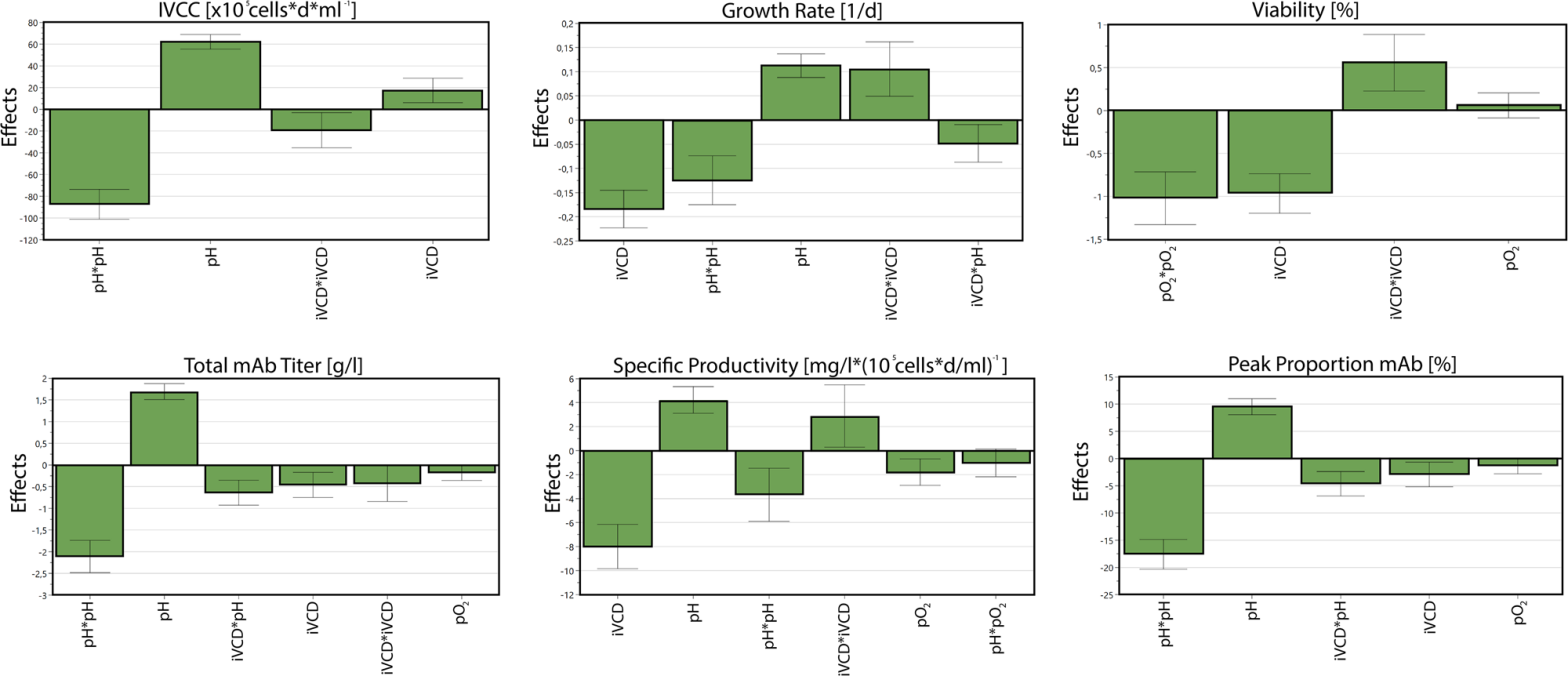
Factor effect plots for the studied responses. Factor squares and interactions are combined with a star. Only significant factors and interactions are considered and displayed in descending order for each response

In order to evaluate the experimental data, a statistical model was calculated using MLR. Interpretation of the model led to specific conclusion for factor effects and interactions for the studied parameters. In this process, factors and factor combinations that include zero in their confidential intervals were considered as not significant and hence removed from the respective model. Figure 4 shows the plotted main effects for the examined responses.

Overall, the factors with the highest impact on most of the selected responses were the culture pH set point and the initial VCD, both as linear effects (pH; iVCD), nonlinear effects (pH*pH; iVCD*iVCD) as well as interaction (iVCD*pH).

The initial VCD showed considerable influence on the growth rate, viability, and the specific productivity with negative coefficients, meaning a lower iVCD resulted in improved responses. Its quadratic effect with positive coefficients for most responses besides the peak proportion showed the nonlinearity of the correlation. Those effects can be explained by faster nutrient consumption and limitation with higher initial VCDs.

The pH showed reversed coefficients for most responses, resulting in a nonlinear correlation. With higher pH values increasing growth rates, integral viable cell concentrations, mAb titers, and purities were observed. Furthermore, small but significant interaction of iVCD and pH with negative coefficients could be found. This shows that a lower VCD in combination with a higher pH set point results in improved quality attributes.

In contrast to the other responses, the viability is mainly influenced by dissolved oxygen and the initial VCD. While the dissolved oxygen has significant effects only as quadratic factor with negative coefficients, the initial VCD showed a correlation comparable to the other responses. Those influences on the end viability highlight the importance of the initial VCD, since it impacted the response throughout the whole process.

Accuracy of the regression models was verified by analyzing the corresponding model statistics. The R‐squared (R^2^) term is the fraction of the variation of the response explained by the model, while the adjusted R‐squared (R^2^_adj_) term is adjusted for the degrees of freedom of the model. Values over 0.5 for these terms show high model significance. Statistical model accuracy of future predictions is estimated by the Q‐squared (Q^2^) term. Values for Q^2^ should exceed 0.1 for a significant model and 0.5 for good model. The model validity checks for diverse model problems. A value less than 0.25 indicates statistically significant model problems, such as presence of outliers, incorrect model terms or transformation problems. The reproducibility compares the variation of the center point replicates to the overall variability, with a value over 0.5 insuring high model reproducibility. Table 2 summarizes the model statistics for the analyzed parameters.

**TABLE 2:** Summarized model statistics for the studied responses. R^2^ representing the model significance, Q^2^ representing the predictive power of the model, model validity representing possible model problems and the reproducibility representing the center point variation compared to the overall variability

	R^2^	R^2^ adj.	Q^2^	Model validity	Reproducibility
Growth rate	0.88	0.86	0.83	0.92	0.82
IVCC	0.96	0.95	0.93	0.63	0.97
Viability	0.81	0.79	0.73	0.97	0.65
Total mAb Titer	0.96	0.95	0.93	0.88	0.95
Specific productivity	0.87	0.84	0.79	0.95	0.76
Peak proportion	0.94	0.93	0.90	0.67	0.96

Analysis of the shown model statistics confirmed the significance, validity, and high reproducibility of all used regression models. The viability model showed the lowest values for R^2^, R^2^ adjusted, Q^2^ and the reproducibility, which are yet high enough for solid model interpretation. The overall variability in the measured viabilities compared to the center point runs was rather low, explaining the reproducibility value of 0.65 and influencing the overall model significance. Yet, the high model validity value for the viability of 0.97 insured that no model problems or outliers were present. All other models showed overall high values of over 0.8 for R^2^ and R^2^ adjusted, validating the models significance. Furthermore, the predictive power for future experiments was confirmed by the high Q^2^ values of mostly over 0.8. Same applies for the model validity and reproducibility with values over 0.6 and 0.75, respectively.

**FIGURE 5:**
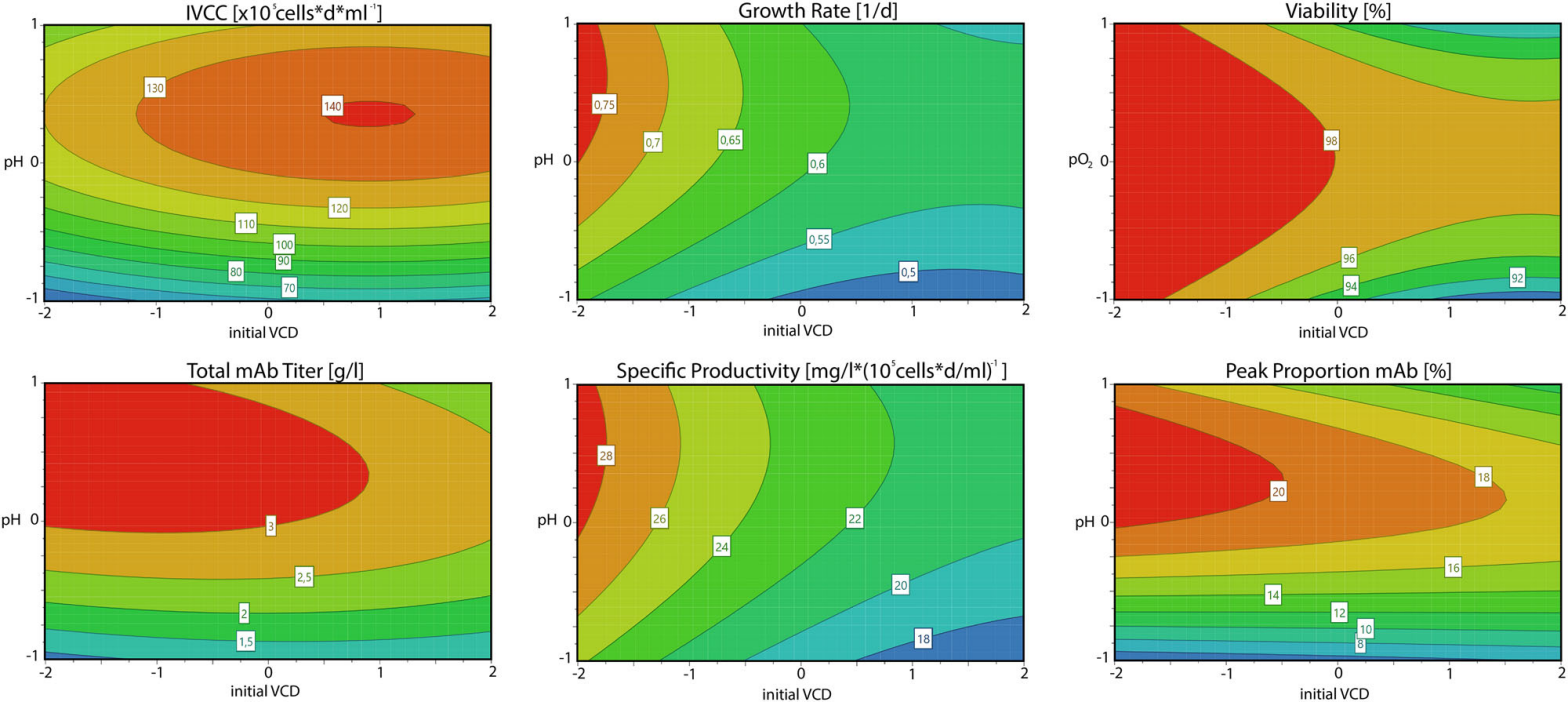
Response contour plots representing the interaction effects of different factors on the studied process responses

After validation of the computed regression models, the findings were extended by investigation of the resulting response counter plots, depicted in Figure 5. A response contour plot provides a two‐dimensional interpretation of the predictors and their respective response values. Using the contour plots it was possible to further investigate factor effects and interactions as well as determine desirable operating conditions.

The IVCC as well as the total mAb titer contour plot confirms the observed pH effect, with an optimum between 0 and 1 conditions. As expected, a higher initial viable cell density led to an increased IVCC with an optimum around 1 condition. However, this effect was counteracted by the improved growth rate for low initial VCDs. The contour plot for the growth rate and the specific productivity responses shows an optimum for a minimal initial VCD and an increased pH set point. Furthermore, it depicts the nonlinear effect of the pH and the lower robustness against variations in the initial VCD.

For the viability response the initial VCD and the dissolved oxygen were used for the response contour plot. Again, lower initial VCD were shown to be beneficial for high culture viabilities throughout the entire process, while the dissolved oxygen showed an optimum around 0 condition, especially for higher initial VCDs. The peak proportion contour plot shows similar effects for the pH as described for the other responses. Yet, the initial VCD effect shows similarities to the viability plot, implying correlations between those responses. This can be explained by host cell proteins and other impurities that are released to the cultivation broth during cell death.

**FIGURE 6:**
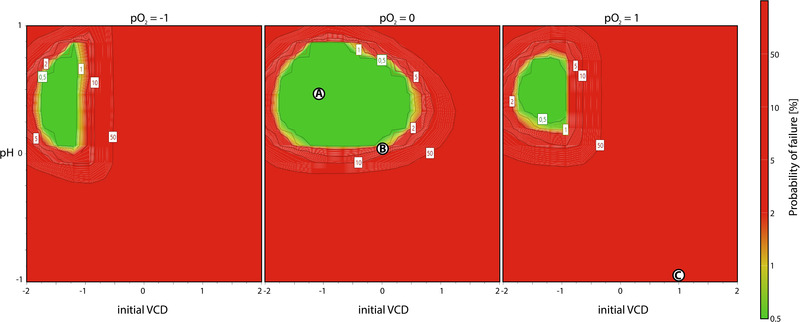
Determined design spaces for the mAb production process with color coded probability of failure for the assessed response specifications. Parameter conditions for the verification runs are marked as A (optimum), B (standard), and C (impaired)

In order to combine the analyzed responses and visualize the experimental design region in which all response specifications are fulfilled with given probabilities, a design space was calculated. Specifications for each response were accounted during the process development and adjusted with the risk assessment as well as the process knowledge acquired during this study. The resulting design space for the factors initial VCD, pH, and dissolved oxygen is shown in Figure 6. The green area marks a robust design space with a low probability for possible process failures.

The design space was determined around –1 level for the initial VCD and between 0 and 1 level for the pH set point. Increase as well as decrease in the dissolved oxygen decreased the design space in its size, shifting it toward a lower initial VCD. Therefore, a process with insufficient pO_2_ control should be inoculated with a lower viable cell density, while the pH effect was shown to be more robust above the center point. Using the design space new optimal set points for the pH with 7.2 and the initial VCD with 0.2 × 106 cells/mL could be specified. Thereby, robust cell growth and viability as well as optimal mAb production with minimal impurities can be targeted for the studied process.

**FIGURE 7:**
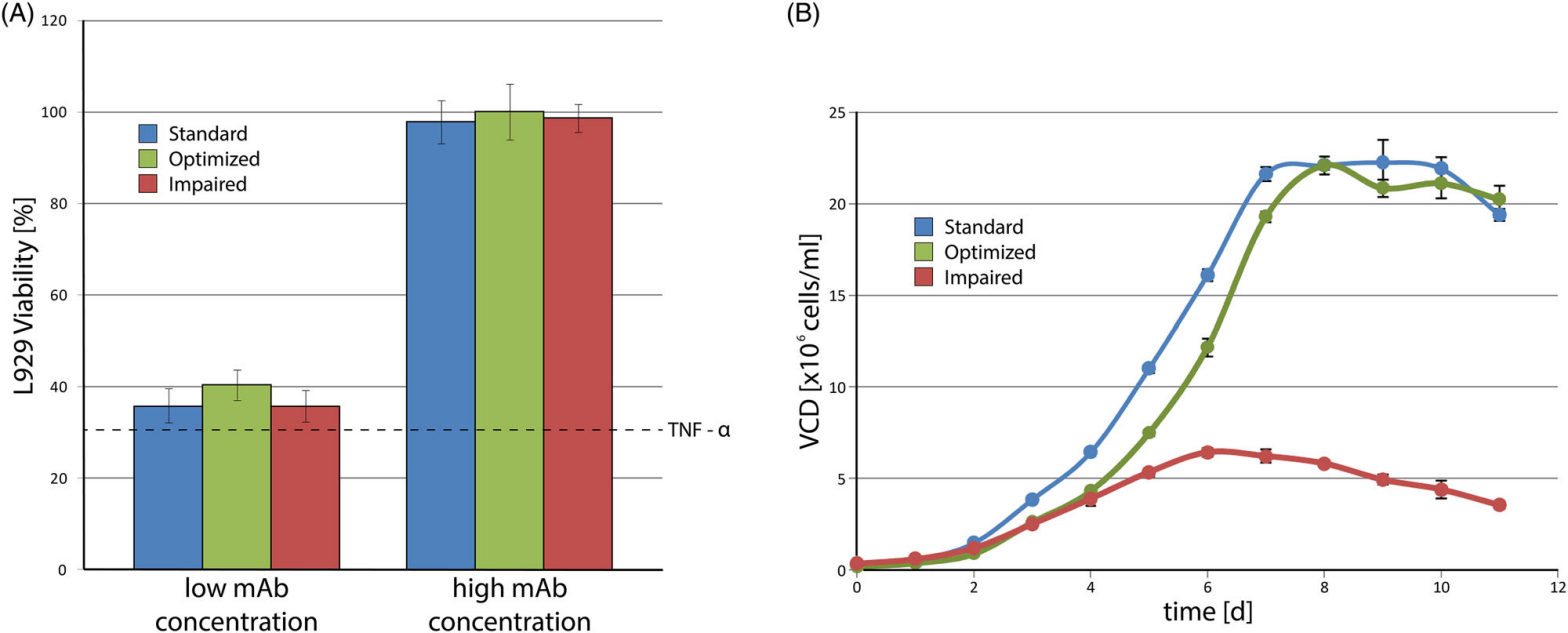
(A) Bioactivity assay for mAb samples of the standard (blue), optimized (green) and impaired (red) runs in two different concentrations (2.5 ng/mL and 50 ng/mL). Cell viability without added antibody is marked by the dashed TNF‐α line. (B) Growth curves of the standard (blue), optimized (green), and impaired (red) runs

The established optimum A (pO_2  _=  60%; pH  =  7.2; iVCD  =  0.2 × 10 [6]), the standard run B (pO_2  _=  60%; pH =  7.1; iVCD  =  0.3 × 106) as well as a run with impaired conditions C (pO_2  _=  80%; pH  =  6.9; iVCD  =  0.4 × 10 [6]) were repeated in three‐fold determination, which confirmed the findings for all assessed responses. Corresponding growth curves are depicted in Figure 7, while the measured responses are summarized in the supplements. While the standard run showed a slightly higher mAb titer and IVCC, its productivity was lower compared to the optimized run. The impaired conditions caused lower cell growth and viabilities, ultimately resulting in a poor mAb titer with larger proportions of host cell proteins. These verifications of the conducted DoE underline the importance of the established process control space. The correlation of end viability and the peak proportion of the product is especially important for the following downstream process.

Likewise, the bioactivity of the produced antibody is an important critical quality attribute. In order to investigate this, the mAb samples produced in the verification runs (A, B and C) were used for a cell based bioactivity assay. This assay is based on the antibody property to bind and inactivate the cytotoxic antigen TNF‐α. By that, the viability of the used L929 cells represents the bioactivity of the antibodies for given concentrations. Results of the assay are shown in Figure 7, in which the dashed TNF‐α line represents cell viability without added antibody.

The applied concentrations were chosen from a previously conducted calibration curve with standard mAb. As expected, the low concentration only showed a small increase in cell viability compared to the pure TNF‐α experiment. The high concentration prevented the apoptotic effect and thereby the decrease in cell viability by binding the applied antigen. Only insignificant differences between the separate runs were observed, with the optimized run showing the highest bioactivity.

These results indicate that the investigated cultivation conditions during the production process have no significant impact on the bioactivity of the produced antibody. By that, the focus must be set on the intermediate quality attributes like high mAb production with special attention to the cell maintenance, in order to ensure low amounts of impurities. This target can be reached by precise process analytics and control based on the established design space.

## 4 CONCLUDING REMARKS

The fundamental concepts of QbD were tested and implemented in the established mAb production process of a CHO cell cultivation. Thereby, this work builds upon previous findings in the inoculum expansion and marks the second step toward a holistic process characterization. Process parameters were compiled and sorted in an Ishikawa‐diagram. Using the FMAE tool, the general risk potential for each parameter was assessed and ranked based upon their probability, severity and detectability. In order to investigate the effect and interaction of the intermediate critical process parameters a complex design of experiments approach was conducted. The experiments were performed using the ambr®15 micro bioreactor platform, aiming for optimal process parameter control and high comparability of the acquired cultivation data. Multivariate data analysis was used to fit a mathematical model and calculate parameter effects and interactions for each respective response. Culture pH and iVCD were determined as nonlinear key process parameters with interaction effects. The observed effects of the initial VCD confirmed the findings for the initial passage VCD during the inoculum expansion studies and its importance on the nutrient consumption and thereby the process control.

All responses and parameter effects were used to establish a design space in which sufficient cell growth as well as antibody production is ensured. In conclusion, the pH should be controlled around 7.2, while the initial VCD should be lowered to 0.2 × 10 [6] cells/mL. The dissolved oxygen should be controlled around the 60% set point. Changes in the N‐1 duration showed only small effects on the process, meaning that a delay in inoculation for less than 2 days can be balanced out with optimal control of the other process parameters. Furthermore, no significant differences in the product quality, namely the bioactivity, were observed for changing cultivation conditions, meaning the priority in quality control for the studied process should be set on high cell maintenance as well as optimal mAb production.

This case study for the implementation of QbD strategies in the mAb production illustrates the suitability of the ambr®15 micro bioreactor system for complex DoE approaches with sufficient process control and high reproducibility. Furthermore, this study highlights the importance of the established Design Space for robust process optimization during the process development. In order to apply the general QbD principles on the entire process, further studies will be focused on the cell removal and the downstream part. Therefore, the correlation of viability and product proportion to total protein as well as other quality attributes will be investigated using different cell removal methods.

## CONFLICT OF INTEREST

We confirm that all corresponding authors agree with the submission and publication of this paper and that there is no conflict of interest concerning financial and personal relationships. The manuscript does not contain neither experiments using animals nor human studies. Furthermore, we confirm that the article has not been published previously by any of the authors and is not under consideration for publication elsewhere at the time of submission.

## Supporting information




Supporting Information

## Data Availability

The data that supports the findings of this study are available in the supplementary material of this article.
