# Supplementary material for: Optimization of a mAb production process with regard to robustness and product quality using quality by design principles
Source: Eng Life Sci. 2022 Jun 3;22(7):484–94. doi: 10.1002/elsc.202100172 (PMC9288990; doi:10.1002/elsc.202100172)
Supplement: Supplementary file 1 — Supporting Information [file ELSC-22-484-s001.docx]

**Supplements – Optimization of a mAb production process with regard to robustness and product quality using Quality by Design principles**

**Table S1.** Numerical coding of varied process parameters.

| Process Parameter | -2 | -1 | 0 | 1 | 2 |
| --- | --- | --- | --- | --- | --- |
| pH | - | 6.9 | 7.1 | 7.3 | - |
| Initial VCD [x10^5^ cells/ml] | 1 | 2 | 3 | 4 | 5 |
| Dissolved oxygen [%] | - | 40 | 60 | 80 | - |
| N-1 duration [d] | - | 2 | 3 | 4 | - |

**Table S2.** Experimental set up and responses for the DoE.

| Experiment number | | Initial VCD | pH | | pO_2_ | | N-1 duration | Growth rate | IVCC | | Viability | Total mAb titer | Specific mAb titer | Peak proportion |
| --- | --- | --- | --- | --- | --- | --- | --- | --- | --- | --- | --- | --- | --- | --- |
| 1 | 3 | | 7.1 | 60 | | 3 | | 0.61 | | 127.9 | 98.3 | 3.24 | 25.3 | 17.79 |
| 2 | 1 | | 6.9 | 40 | | 2 | | 0.57 | | 33.4 | 97.9 | 0.84 | 25.2 | 3.78 |
| 3 | 2 | | 6.9 | 40 | | 4 | | 0.57 | | 48.3 | 96.4 | 1.04 | 21.5 | 4.75 |
| 4 | 4 | | 6.9 | 40 | | 2 | | 0.48 | | 58.6 | 87.5 | 1.05 | 17.9 | 5.5 |
| 5 | 5 | | 6.9 | 40 | | 4 | | 0.50 | | 70.6 | 85.7 | 1.2 | 17.0 | 6.32 |
| 6 | 1 | | 7.3 | 40 | | 4 | | 0.78 | | 107.7 | 99.4 | 3.17 | 29.4 | 18.07 |
| 7 | 2 | | 7.3 | 40 | | 2 | | 0.67 | | 113.7 | 95.5 | 1.92 | 16.9 | 10.51 |
| 8 | 4 | | 7.3 | 40 | | 4 | | 0.60 | | 121.5 | 96.1 | 3.07 | 25.3 | 15.09 |
| 9 | 5 | | 7.3 | 40 | | 2 | | 0.53 | | 94.4 | 84.7 | 1.96 | 20.8 | 10.13 |
| 10 | 1 | | 6.9 | 80 | | 4 | | 0.63 | | 31.4 | 99 | 0.93 | 29.7 | 4.11 |
| 11 | 3 | | 7.1 | 60 | | 3 | | 0.66 | | 133.3 | 98.6 | 2.77 | 20.8 | 4.09 |
| 12 | 2 | | 6.9 | 80 | | 2 | | 0.59 | | 60.74 | 95.6 | 1.23 | 20.3 | 6.42 |
| 13 | 4 | | 6.9 | 80 | | 4 | | 0.49 | | 58.7 | 92.4 | 0.91 | 15.5 | 4.37 |
| 14 | 5 | | 6.9 | 80 | | 2 | | 0.45 | | 66.5 | 93.3 | 1.18 | 17.8 | 6.49 |
| 15 | 1 | | 7.3 | 80 | | 2 | | 0.76 | | 106.7 | 99.1 | 2.93 | 27.5 | 15.39 |
| 16 | 2 | | 7.3 | 80 | | 4 | | 0.57 | | 54.8 | 87.5 | 0.97 | 17.7 | 4.84 |
| 17 | 4 | | 7.3 | 80 | | 2 | | 0.54 | | 103.6 | 97.9 | 1.99 | 19.2 | 9.21 |
| 18 | 3 | | 7.1 | 60 | | 3 | | 0.66 | | 142.1 | 99.1 | 3.4 | 23.9 | 20.14 |
| 19 | 5 | | 7.3 | 80 | | 4 | | 0.53 | | 106.2 | 86.8 | 1.9 | 17.9 | 9.02 |
| 20 | 3 | | 7.1 | 60 | | 3 | | 0.58 | | 126.8 | 96.9 | 3.02 | 23.8 | 18.24 |
| 21 | 3 | | 6.9 | 40 | | 2 | | 0.49 | | 62.0 | 89.9 | 1.24 | 20.0 | 6.39 |
| 22 | 3 | | 7.3 | 80 | | 4 | | 0.61 | | 131.4 | 93.4 | 2.76 | 21.0 | 15.68 |
| 23 | 3 | | 7.1 | 40 | | 3 | | 0.62 | | 139.7 | 93 | 3.06 | 21.9 | 18.86 |
| 24 | 3 | | 7.3 | 40 | | 2 | | 0.61 | | 123.7 | 93.9 | 3.09 | 25.0 | 19.16 |
| 25 | 3 | | 7.3 | 60 | | 3 | | 0.62 | | 137.0 | 96.8 | 3.16 | 23.1 | 18.58 |
| 26 | 3 | | 7.3 | 40 | | 4 | | 0.57 | | 116.7 | 92.3 | 2.78 | 23.8 | 14.83 |
| 27 | 3 | | 7.1 | 80 | | 3 | | 0.58 | | 133.0 | 95.5 | 2.92 | 22.0 | 17.54 |
| 28 | 3 | | 7.1 | 60 | | 2 | | 0.57 | | 130.2 | 98.3 | 2.93 | 22.5 | 18.81 |
| 29 | 3 | | 7.1 | 60 | | 4 | | 0.61 | | 124.2 | 97.7 | 2.86 | 23.0 | 18.24 |
| 30 | 3 | | 7.1 | 60 | | 3 | | 0.61 | | 138.9 | 96.4 | 3.11 | 22.4 | 20.71 |
| 31 | 3 | | 6.9 | 40 | | 4 | | 0.49 | | 57.6 | 94.2 | 1.08 | 18.7 | 5.9 |
| 32 | 3 | | 7.3 | 80 | | 2 | | 0.59 | | 115.1 | 93.9 | 2.72 | 23.6 | - |
| 33 | 3 | | 6.9 | 60 | | 3 | | 0.45 | | 54.3 | 84 | 0.93 | 17.1 | 4.65 |
| 34 | 3 | | 7.1 | 60 | | 3 | | 0.59 | | 133.2 | 98.1 | 3.0 | 22.5 | 19.3 |
| 35 | 3 | | 6.9 | 80 | | 2 | | 0.46 | | 54.5 | 91.7 | 0.97 | 17.8 | 4.87 |
| 36 | 3 | | 6.9 | 80 | | 4 | | 0.45 | | 53.0 | 93.6 | 1.04 | 19.6 | 5.33 |

**Table S3.** Parameter limits for the design space calculation

|  | Growth rate | IVCC | Viability | Total mAb titer | Specific mAb titer | Peak proportion |
| --- | --- | --- | --- | --- | --- | --- |
| Parameter limit | 0.55 | 110 | 95 | 2.9 | 20 | 16 |
| Target | 1 | 160 | 100 | 3.5 | 30 | 25 |

**Table S4.** Responses for the verification runs

| Experiment | Growth rate | IVCC | Viability | Total mAb titer | Specific mAb titer | Peak proportion |
| --- | --- | --- | --- | --- | --- | --- |
| Standard 1 | 0.60 | 139.6 | 97.3 | 3.49 | 25.0 | 17.64 |
| Standard 2 | 0.63 | 137.8 | 97.1 | 3.13 | 22.7 | 17.0 |
| Standard 3 | 0.60 | 134.7 | 98.5 | 3.43 | 25.4 | 16.35 |
| Optimized 1 | 0.66 | 115.6 | 95.4 | 3.0 | 26.0 | 15.93 |
| Optimized 2 | 0.67 | 124.3 | 98.1 | 3.33 | 26.8 | 15.4 |
| Optimized 3 | 0.65 | 124.7 | 97.8 | 3.16 | 25.3 | 15.85 |
| Impaired 1 | 0.41 | 46.5 | 75.4 | 0.68 | 14.6 | 2.77 |
| Impaired 2 | 0.39 | 38.1 | 77.9 | 0.64 | 16.8 | 2.96 |
| Impaired 3 | 0.42 | 45.8 | 80.7 | 0.69 | 15.1 | 2.89 |
